# Deep Learning for Per-Fraction Automatic Segmentation of Gross Tumor Volume (GTV) and Organs at Risk (OARs) in Adaptive Radiotherapy of Cervical Cancer

**DOI:** 10.3389/fonc.2022.854349

**Published:** 2022-05-18

**Authors:** Adrian L. Breto, Benjamin Spieler, Olmo Zavala-Romero, Mohammad Alhusseini, Nirav V. Patel, David A. Asher, Isaac R. Xu, Jacqueline B. Baikovitz, Eric A. Mellon, John C. Ford, Radka Stoyanova, Lorraine Portelance

**Affiliations:** Department of Radiation Oncology, Sylvester Comprehensive Cancer Center, Miller School of Medicine, University of Miami, Miami, FL, United States

**Keywords:** MRI-guided radiotherapy, cervical cancer, radiotherapy, adaptive radiotherapy, deep learning, convolutional neural networks

## Abstract

**Background/Hypothesis:**

MRI-guided online adaptive radiotherapy (MRI-g-OART) improves target coverage and organs-at-risk (OARs) sparing in radiation therapy (RT). For patients with locally advanced cervical cancer (LACC) undergoing RT, changes in bladder and rectal filling contribute to large inter-fraction target volume motion. We hypothesized that deep learning (DL) convolutional neural networks (CNN) can be trained to accurately segment gross tumor volume (GTV) and OARs both in planning and daily fractions’ MRI scans.

**Materials/Methods:**

We utilized planning and daily treatment fraction setup (RT-Fr) MRIs from LACC patients, treated with stereotactic body RT to a dose of 45-54 Gy in 25 fractions. Nine structures were manually contoured. MASK R-CNN network was trained and tested under three scenarios: *(i)* Leave-one-out (LOO), using the planning images of N- 1 patients for training; *(ii)* the same network, tested on the RT-Fr MRIs of the “left-out” patient, *(iii)* including the planning MRI of the “left-out” patient as an additional training sample, and tested on RT-Fr MRIs. The network performance was evaluated using the Dice Similarity Coefficient (DSC) and Hausdorff distances. The association between the structures’ volume and corresponding DSCs was investigated using Pearson’s Correlation Coefficient, r.

**Results:**

MRIs from fifteen LACC patients were analyzed. In the LOO scenario the DSC for Rectum, Femur, and Bladder was >0.8, followed by the GTV, Uterus, Mesorectum and Parametrium (0.6-0.7). The results for Vagina and Sigmoid were suboptimal. The performance of the network was similar for most organs when tested on RT-Fr MRI. Including the planning MRI in the training did not improve the segmentation of the RT-Fr MRI. There was a significant correlation between the average organ volume and the corresponding DSC (r = 0.759, p = 0.018).

**Conclusion:**

We have established a robust workflow for training MASK R-CNN to automatically segment GTV and OARs in MRI-g-OART of LACC. Albeit the small number of patients in this pilot project, the network was trained to successfully identify several structures while challenges remain, especially in relatively small organs. With the increase of the LACC cases, the performance of the network will improve. A robust auto-contouring tool would improve workflow efficiency and patient tolerance of the OART process.

## Introduction

Radiotherapy (RT) targets can be mobile, deformable structures (1). In non-adaptive RT, the target for fractionated treatment is defined based on a single pretreatment CT or MRI planning scan with a security margin added to account for anatomic variability (2, 3). The security margin must be large enough to prevent a geographic miss, which often translates into the inclusion of adjacent normal tissue that is vulnerable to radiation-related toxicity in the treated volume (4). An attractive alternative is Magnetic Resonance Image-guided online adaptive RT (MRI-g-OART). When an MRI-g-OART approach is used, daily MR setup scans provide accurate soft-tissue visualization of the target and organs-at-risk (OARs), allowing physicians to modify the original treatment plan based on the anatomy of the day. This approach has been shown to improve target coverage and OARs sparing compared to non-adaptive techniques, improving the therapeutic index of RT for various malignancies (5–10).

For patients with locally advanced cervical cancer (LACC) undergoing external beam radiotherapy (EBRT), changes in bladder and rectal filling contribute to large inter-fraction target volume motion (11). Conventional strategies to address this include expanding the planning target volume (PTV) by up to 2 cm, potentially exposing the bladder, rectum and bowel to elevated doses (12). Definitive RT for LACC using CT-based non-adaptive techniques has been associated with high incidences of early (27%) and late (10%) toxicity (13). In the acute setting, up to 25% of patients experience at least grade 3 gastrointestinal (GI) toxicity and 10% at least grade 3 genitourinary (GU) toxicity. Eighteen percent of patients require treatment interruptions of more than seven days due to the severity of acute symptoms (13). MRI-g-OART promises more conformal dose delivery than the expanded PTV approach, with the potential to improve clinical outcomes by limiting treatment interruptions associated with radiation-related toxicity (14–16).

Technical challenges to OART are not negligible. MRI-g-OART is time-intensive, requiring delineation of OARs near the target volume by the supervising radiation oncologist or a dedicated trained technologist while the patient remains immobile on the treatment table (17). Delays in the adaptive process can challenge patients’ tolerance of OART and increase the likelihood of anatomic changes during the interval between image acquisition and completion of radiation delivery (17). Various strategies are under investigation to improve workflow efficiency, including the use of artificial intelligence (AI) deep learning (DL) techniques such as convolutional neural networks (CNN), already applied successfully in diagnostic imaging classification (18, 19).

In this study, we propose using the MASK R-CNN architecture for segmenting the GTV and OARs in a LACC MRI-g-OART treatment scenario. Generally, CNNs are used in image classification systems, where the system is trained on a collection of images and their labels, and then used to classify unseen images into their corresponding categories. The MASK R-CNN extends this into instance segmentation – where the network detects individual objects in the image, generates a mask to segment the object from the rest of the image, and assigns a class to the segmented object (20).

In the case of MRI data, the MASK R-CNN is used to segment and classify the GTV and OARs within the images. The MASK R-CNN architecture includes multiple sub-CNNs. First, the image is fed into a CNN backbone which generates feature maps. These feature maps are then provided to a region proposal network (RPN) which proposes regions that may contain objects of interest. At the second stage, the MASK R-CNN network predicts classes for each region of interest and a refined object mask. The MASK R-CNN outputs the refined masks of the classified objects, working as an automatic segmentation and classification algorithm.

We hypothesized that (*i*) MASK R-CNN can quickly and accurately segment GTVs and OARs in MRI-g-OART of LACC; (*ii*) MASK R-CNN, trained on the initial planning MRIs can segment images of OART fractions of an “unseen” patient, i.e. one whose initial RT planning MRI were not used to train the system; and *(iii)* augmentation of trained MASK R-CNN with the unseen patient’s initial planning MRI (“transfer learning”) can improve the segmentation of subsequent OART fractions.

## Materials and Methods

### Study Cohort, MRI Acquisition and Contouring

MRI studies were selected from patients treated for LACC on the MRIdian^®^ system (ViewRay, Inc., Mountain View, CA) and enrolled in our Institutional Review Board (IRB) approved registry. Patients were treated using stereotactic body radiation therapy (SBRT) to a dose of 45-54 Gy in 25 fractions.

All MRIs were acquired on a 0.35T MRIdian combination MRI-g RT system. The MRI sequence used was a balanced steady-state free precession technique (True FISP), providing T2/T1-weighted contrast. Studies from the planning MR and daily MR image guidance acquired before every fraction were used. The planning MRIs were acquired with voxel dimensions of 1.5 × 1.5 × 1.5 mm^3^, and the following pulse sequence acquisition parameters: TR/TE = 3/1.27ms, flip angle = 60, bandwidth = 604 Hz/pixel, FOV = 501 × 300 × 360 mm (in left-right, anterior-posterior, and head-foot directions), and matrix size 334 × 200 × 240. The MRI of the treatment fractions were acquired with voxel dimensions of 1.5 × 1.5 × 3.0 mm^3^ and matrix size 360 × 310 × 144.

**Figure 1:**
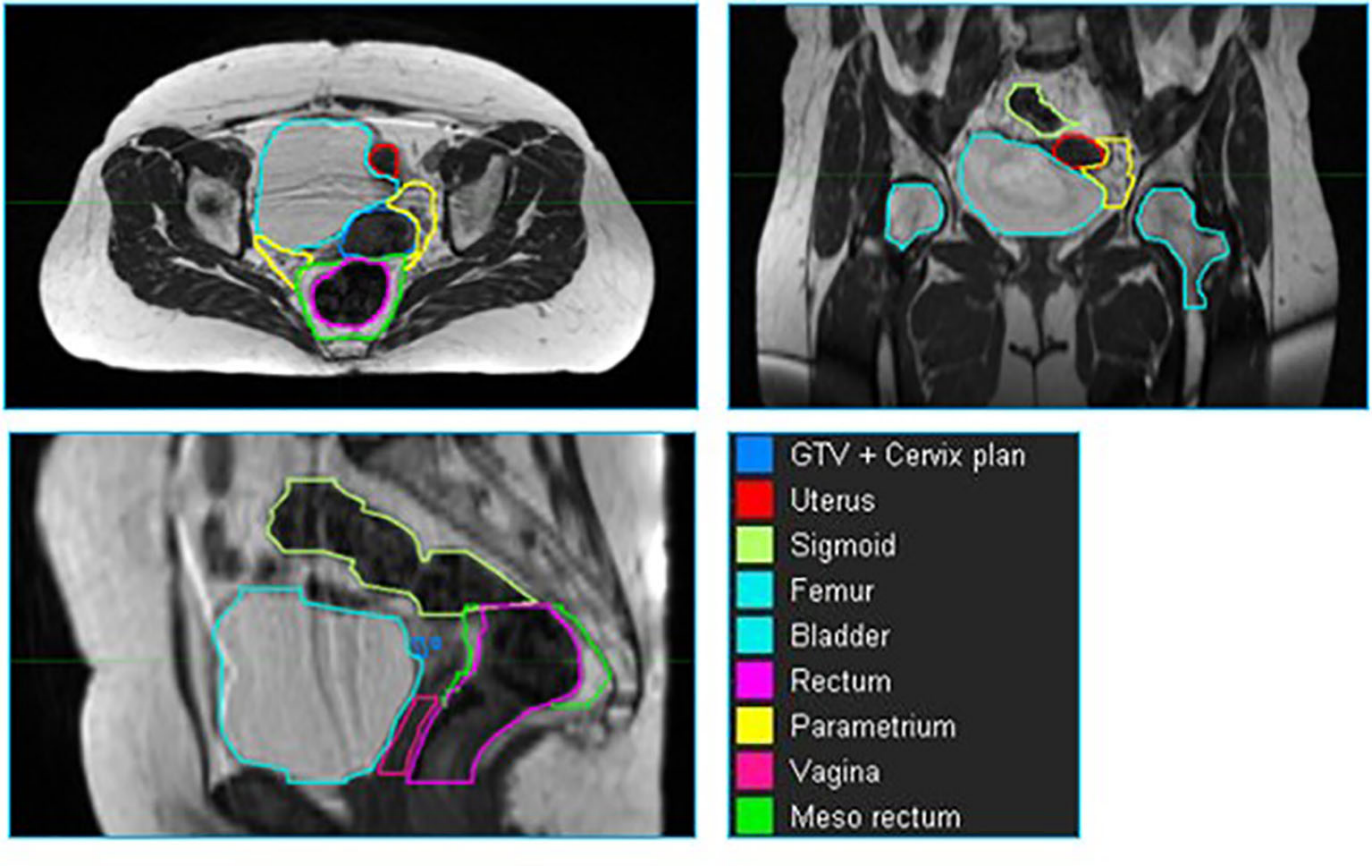
An example of a cervix case provided to the neural network for training. The individual volumes are depicted in different colors.

Across all patients, nine structures (GTV + cervix, uterus, parametrium, sigmoid, bladder, vagina, femur, rectum and mesorectum) were contoured for each patient in MIM. The volumes were delineated within a ROI from the top of the first sacral vertebra (S1) to the bottom of the lesser femoral trochanters. The contours from the treatment plan were used as a basis of the organ segmentation. The contours were checked and refined by radiation oncologists specialized in the treatment of gynecological cancers. Examples of these contours are shown in **Figure 1**.

### Preprocessing of Images and Contours

A preprocessing pipeline, implemented in Python, has been developed to prepare the raw MR images and contours as inputs for the network. The image intensities were normalized to the interval of [0,1] by scaling the 1^st^ and 99^th^ percentiles of the original image intensities. From the whole MRI volume of data, only slides with at least one manual contour were used for network training. The dataset was augmented by flipping the images on the sagittal axis in order to increase the number of training examples. These images were saved into the PNG format at the original 2D resolution of the source MR.

### MASK R-CNN: Training, Validating and Testing

The MASK R-CNN architecture (20) implemented in Tensorflow (21) was used for automated image segmentation and classification. Input images were resized from their native resolutions to an overall size of 512 × 512 pixels per slice. In addition to our data augmentation process, MASK R-CNN implements a layer of data augmentation. By random selection, some of the images were altered with up to two different data augmentation techniques selected from vertical flips, horizontal flips, rotations, multiplication, or Gaussian blur. The images were then fed into the network for training.

Initially, the network weights were loaded from a trained ImageNet model (22). Our network’s training parameters were configured as described by Johnson (23).

The network is optimized through stochastic gradient descent (SGD) with the following hyperparameters: learning rate α = 0.001, momentum of 0.9 and decay of 10^-6^. The training was performed using a batch size of 16 images. MASK R-CNN uses several different loss functions to evaluate and compute weights for the overall network: The RPN and the classifier head use cross-entropy loss and SoftMax loss, respectively, with smoothed L1 loss to refine their anchors and bounding boxes. The mask generator uses binary cross-entropy loss to refine its mask outputs. The individual loss functions are computed as:


$$\mathrm{Cross-entropy}\;\mathrm{loss}:\;-\sum_{c=1}^{M}y_{o,c}\text{ln}\left(p_{o,\;c}\right)$$

where *M* is the number of classes, *y_o,c_* is a binary (1 or 0) indication if class label *c* is the correct classification for observation *o*, and *p* is the probability observation *o* is in class *c*.


$$\mathrm{Softmax}\;\mathrm{cross-entropy}\;\mathrm{loss}:-\sum_{i=1}^{n}\sum_{c=1}^{M}y_{i_{o,c}}\text{In}(p_{i_{o,c}})$$

where *n* is the batch size, *M* is the number of classes, *yi_o,c_* is a binary (1 or 0) indication if class label *c* is the correct classification for observation *o*, and *p* is the probability observation *o* is in class *c*.


$$\mathrm{Binary}\;\mathrm{cross-entropy}:-\left(y_{o,c}\ln\left(p_{o,c}\right)+\left(1-y_{o,c}\right)\ln\left(1-p_{o,c}\right)\right)$$

where *y_o,c_* is a binary (1 or 0) indication if class label *c* is the correct classification for observation *o*, and *p* is the probability observation *o* is in class *c*. This is equivalent to the cross-entropy loss formula above in the instance where *M* = 2.


$$Smooth\;L1\;loss:\{\begin{array}{cc} \left|\hat{y}-y\right|, & if\left|\hat{y}-y\right|>\alpha ;\\ \frac{1}{\alpha }{\left(\hat{y}-y\right)}^{2}, & if\left|\hat{y}-y\right|\leq \alpha \end{array}$$

where α is 1, *y* is the predicted output, and ŷ is the target output.

The training was run on a flexible number of epochs, with the stopping criteria defined as three epochs without an improvement in the combined average value of the loss functions in the validation set (10% of the training dataset).

The training was performed on a multi-GPU cluster computer (3 x NVIDIA Quadro RTX 8000, 48 GB memory each). Each model training took approximately 3.5 hours and the automatic segmentation process (inference) takes less than 50s. The system was implemented using Keras (24) and TensorFlow (21) Python libraries.

### Mask R-CNN Output

Classification in Mask R-CNN was carried out *via* parallel prediction of contour masks and class labels, using the ResNet backbone network to determine the most appropriate object class, and then applying the masking branch for that class (20). The output was a binary mask representing an instance segmentation of the detected class. Separately, the network also produced a confidence parameter (between 0 and 1) for the class nominated by the RPN. The image and the accompanying mask were generated at the original resolution of the input, single axial images as described above. To recover the original RT-DICOM structure, we developed custom code to re-assemble the 2D masks into 3D contour volumes, integrated as part of the overall workflow. These RT-DICOMs are platform-agnostic and can be viewed in any RT treatment planning system. The output images may be viewed with any conventional image viewer.

### Experimental Design and Statistical Analysis

The network’s purpose is to generate automatic segmentation of OARs and GTVs for initial RT planning of a new patient and for daily re-contouring at the treatment images for patients receiving OART. Three different scenarios were considered:

- Leave-one-out (LOO) - Training was conducted in LOO schema, whereby the planning images of N- 1 patients were used as training data for the network, and the excluded patient’s planning image was used for testing. In the end, a total of N training sequences were performed, with each patient serving as a training example N- 1 times, and as a test example once.
- RT Fraction (RT-Fr) - We evaluated the network’s effectiveness in contouring images from the treatment fractions of the unseen patient. The network trained in scenario (i) is tested on images from the treatment fractions of the excluded patient.
- Transfer learning - We investigated whether including the planning MRI of the unseen patient as an additional training sample to the trained network would allow the network to perform better on the treatment fraction MRIs for the same patient.

**Figure 2:**
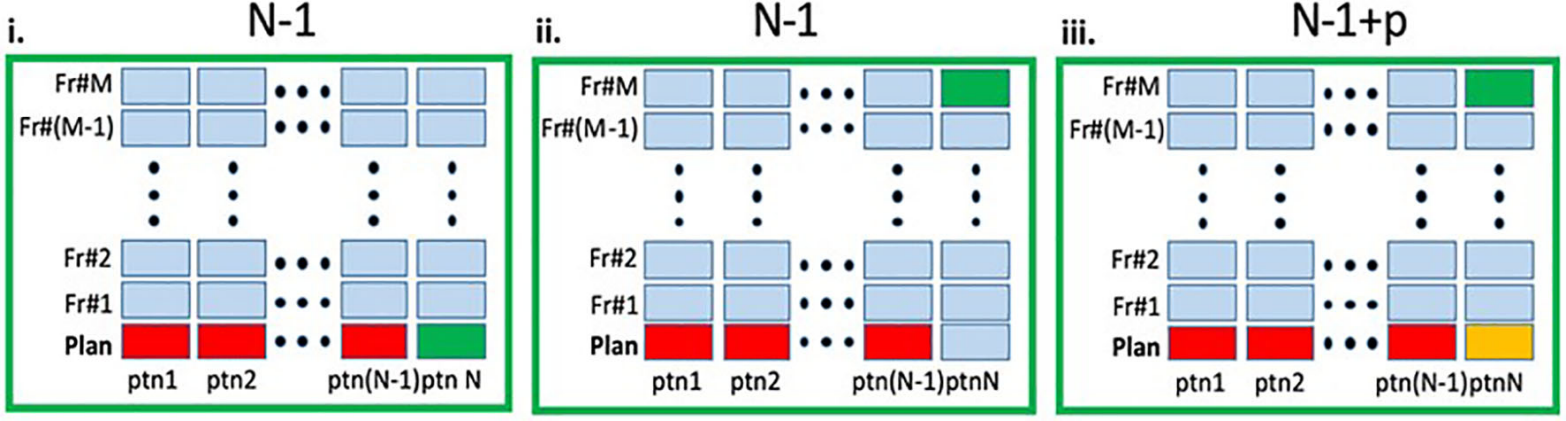
Schematic representation of the experimental design. In each panel the columns represent the MRIs (planning and fraction 1 to M) for a given patient. The entire dataset contain total of N×(M+1) MRIs. i. *Leave-one-out (LOO)*: A deep learning (DL) network, marked as N - 1 was trained on the planning MRIs (red) from N - 1 patients and tested on the planning MRI of the left-out patient (green). ii. *RT-Fr*: The N - 1 network was tested on an MRI from an online adaptable radiotherapy fraction of the "left-out" patient (green). Note that the planning MRI from this patient was not used in the training. iii. *Transfer learning*: The planning MRI for patient N (yellow) is added to the N - 1 network, resulting in N - 1+p network, which is then tested on an MRI from RT-Fr of the "left-out" patient (green).

A schematic representation of the three scenarios can be found in **Figure 2**. For all scenarios detailed above, the overall network performance was assessed *via* summary statistics of the Dice Similarity Coefficient (DSCs) and 95% Hausdorff distances between manual and network-generated contours. We also investigated how the network performance is affected by the volume of the contoured structure by correlating the average volume of the manually contoured organ and the corresponding DSC between manual and network-generated contours using Pearson’s Correlation Coefficient, r.

## Results

**Figure 3:**
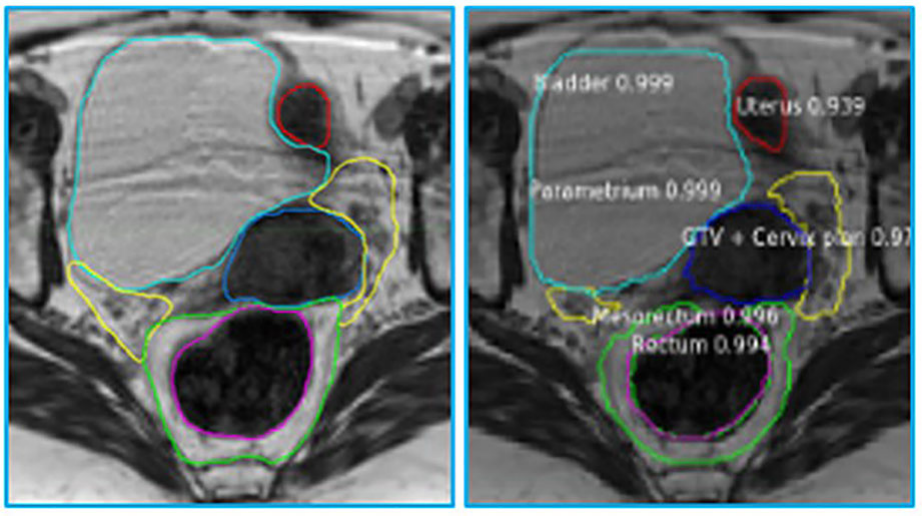
Manual (left) and automatic (right) contours on the original MRI image from a patient with cervical cancer. The contours generated by MASK R-CNN contain a confidence estimate, a number between 0 and 1, with 0 representing mimmum and 1 maximum confidence that the class, assigned to the segmented volume is accurate. Please refer to **Figure 1** legend for color scheme.

The MRIs of fifteen patients (median age 56, range 32 - 71) who received RT for LACC between 2017 through 2018 were analyzed. On average, 83 axial slices per patient were analyzed. **Table 1** shows the obtained DSCs and Hausdorff distances for the segmentation of the GTV and OARs in the three scenarios (**Figure 2**). In the first scenario, fifteen networks were trained from scratch using the MRIs from 14 patients and tested on the “left-out” patient. In this case, the best performance was achieved for Rectum, Femur, and Bladder (DSC > 0.8). The performance was moderate for the Mesorectum, Uterus, Parametrium, and GTV (DSC > 0.6). The results for the Vagina and Sigmoid were suboptimal (DSC ~ 0.4 - 0.5). The performance of the trained network on the left-out patient’s treatment fraction (scenario ii) MRIs markedly improved for the Sigmoid and worsened for the Vagina. The performance of the network on the patient’s treatment fraction after training on their planning MRI improves for the Uterus but deteriorates for the Femur and Vagina. In **Figure 3**, the manual and MASK R-CNN contours on the original MRI image from a patient with LACC are shown.

**Table 1:** Dice Similarity Coefficients (DSC) and Hausdorff distances (HD) (mean ± SD) between the manual and network contours for each of the investigated scenarios.

	Scenario					
	LOO		RT-Fr		Transfer Learning	
	DSC	HD (mm)	DSC	HD (mm)	DSC	HD (mm)
**Mesorectum**	0.62 ± 0.11	2.65 ± 0.89	0.69 ± 0.12	3.13 ± 0.76	0.63 ± 0.11	3.84 ± 1.35
**Rectum**	0.85 ± 0.09	1.18 ± 0.49	0.88 ± 0.07	1.77 ± 0.55	0.85 ± 0.05	1.94 ± 0.76
**Uterus**	0.70 ± 0.23	3.54 ± 3.28	0.69 ± 0.36	3.29 ± 1.44	0.83 ± 0.08	3.50 ± 1.99
**Vagina**	0.41 ± 0.33	2.51 ± 2.00	0.18 ± 0.36	2.14 ± 0.10	0.04 ± 0.07	6.5 ± 0.10
**Parametrium**	0.62 ± 0.09	4.31 ± 2.34	0.58 ± 0.11	4.94 ± 1.02	0.59 ± 0.07	4.72 ± 1.63
**Sigmoid**	0.46 ± 0.26	7.41 ± 5.76	0.69 ± 0.22	8.26 ± 0.98	0.61 ± 0.03	8.26 ± 0.99
**Femur**	0.88 ± 0.06	2.97 ± 1.82	0.76 ± 0.12	1.68 ± 0.25	0.45 ± 0.37	1.68 ± 0.25
**Bladder**	0.81 ± 0.15	3.10 ± 3.57	0.75 ± 0.12	3.01 ± 1.32	0.82 ± 0.09	3.02 ± 1.32
**GTV**	0.67 ± 0.30	2.77 ± 1.73	0.61 ± 0.32	4.34 ± 2.83	0.60 ± 0.32	4.34 ± 2.83

LOO, leave-one-out; RT-Fr, Online Adaptive Radiotherapy Fraction; DSC, Dice Similarity Coefficient; GTV, Gross Tumor Volume; HD, Hausdorff distance.

**Figure 4:**
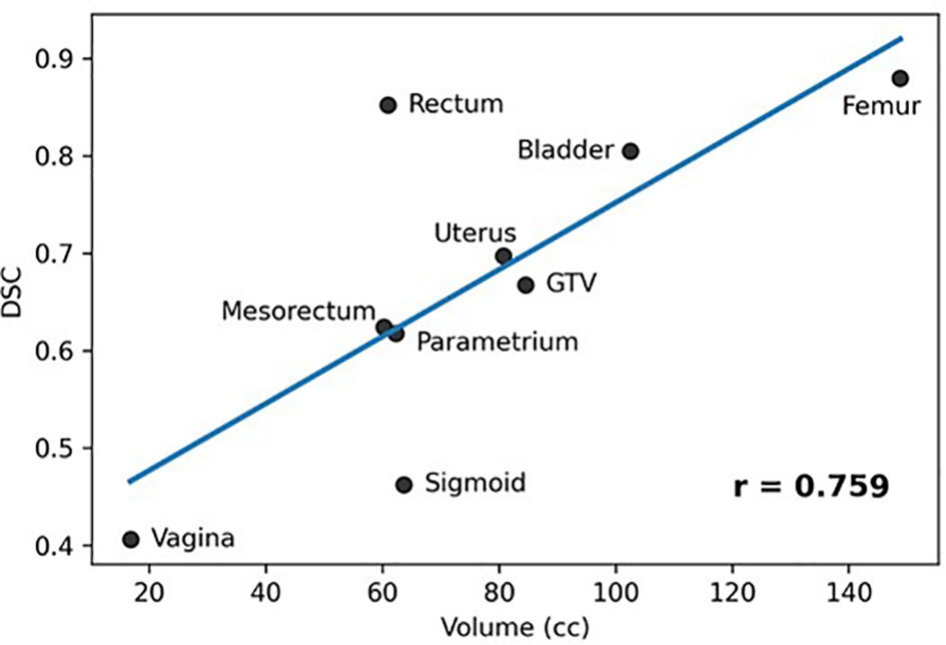
Association between averages of manual stmctures' volumes and corresponding DCS (Pearson Coefficient = 0.759, p = 0.018).

**Figure 4** shows a plot of the average structure volume and the corresponding average DSC of the trained network. As can be seen in the figure, there was a significant association between the average structure’s volume and the corresponding DSC (r = 0.759, p=0.018).

For scenario iii: *Transfer Learning*, we experimented with the number of epochs to train the network on the unseen patient’s planning MRI. We tested a varying number of epochs (20, 40, 80, 100, 200) of transfer learning and evaluated the change in performance on both the unseen and original training datasets. After these trials, we achieved the best overall performance using 20 epochs of transfer learning. In summary, the network’s performance across the original training and validation exams was consistent with the results in **Table 1**.

## Discussion

This study evaluated the performance of the MASK R-CNN network for segmenting the OARs and GTVs in pelvic radiation for LACC. The imaging studies used included MR simulation scans performed in the initial planning phase and daily MR setup scans performed prior to each fraction in MRI-g-OART. Several developments were carried out related to the project: *(i)* the contours of the OARs and GTVs were converted from RT-DICOM to labeled segmentations, *(ii)* DSC and Hausdorff units were implemented to evaluate its performance, and *(iii)* the contours generated from the network were converted into RT-DICOM for transferring to the radiation treatment planning system. The network is universal and accepts images of any dimensions; there is no need for the extended processing often required in other DL approaches.

The network provided segmentations with variable accuracy for the individual organs. Visceral OARS are deformable and mobile, with volumes changing day-to-day based on factors such as stomach contents and stool passing through the intestinal tract. Mobile organs with low-contrast borders such as the Sigmoid presented a serious challenge. On the other hand, the higher contrast of the volume boundaries in organs like the Rectum and Bladder contributed to better segmentation. In these cases, for example, the boundaries are defined by a significant difference in the image intensity relative to the surrounding tissues and the DL contours appear to be smoother and more conformal than manual contours (**Figure 3**). On average, the DSC for the GTV was 0.64, requiring further improvement to be clinically applicable. It should be noted that in current MRI-g-OART workflow, the GTV is prioritized and highly scrutinized by the treating radiation oncologist, a scenario likely to continue regardless of network developments. The association of the poor network performance with the smaller size of an organ (**Figure 4**) explains in part the results related to the Vagina. A difference in only a few pixels between the manual and network contours may have a large impact on the DCS (25). Conversely, the relatively large size may also be a contributing factor for the good performance for Rectum and Bladder.

To the best of our knowledge there are no published reports on segmentation of LACC OARs and GTVs on MRI. Chen et al (26) compared nine methods for segmenting cervical tumors (GTV) on 3D (18)FDG PET images from 50 patients; the highest DSC was 0.84 ± 0.007. The best results achieved by our networks for GTV were comparable DSC = 0.75 ± 0.01. The smaller sample size of the current study and the relatively lower signal-to-noise of MRI compared to PET should be noted. In Fu et al. (18), a CNN network was used to segment OARs: liver, kidney, stomach, bowel and duodenum in the treatment of pancreas, liver, stomach, adrenal gland, and prostate. Despite the significantly larger dataset of MRIs from 120 patients, the DSCs for these five structures were not markedly better. The duodenum was the most challenging structure in their study with DSC of ~0.60. The relatively robust performance of our networks despite training with small datasets is due in part of utilizing MASK R-CNN. Its backbone, the ResNet50 network, is pre-trained with images from the ImageNet database, containing over 14 million images. Instead of starting the training from scratch, the trained “weights” of ImageNet are used by default. This allows the network to be trained satisfactorily on new datasets with few examples.

The network trained for scenario i: LOO on patients’ planning MRIs can be used for the automatic segmentation of OARs and GTVs on an MRI scan in initial RT planning of a new patient. To incorporate these developments into the clinical workflow, the quality of the generated contours must be deemed sufficient by radiation oncologists. While for some organs the results are suboptimal, a process has been created to incorporate the network into our workflow, and to continue its optimization as new datasets become available.

The performance of the network in segmenting the planning MRI (scenario i: LOO) and the daily treatment fraction MRI (scenario ii: RT-Fr) was not markedly different. This second scenario is relevant to offline adaptive planning to account for changes in the tumor size or shape, and especially to online adaptive planning based on the anatomy of the day. In the latter case, fast and robust automated segmentation while the patient is on the table has the potential to decrease treatment time, improving patients’ tolerance of OART and limiting anatomic changes in the interval between image acquisition and radiation delivery. Note that the treatment fraction MRIs were acquired with different sequence parameters. As shown by others (27) and in our work (28, 29), the variability in the data acquisition contribute to the generalization of the network. The fact that the network performance in scenario ii did not deteriorate indicates the generalizability of the approach.

We also investigated whether adding the simulation MRI to the training of the network (scenario iii: Transfer learning) improves the segmentation performance on subsequent fractions. The rationale was to learn the general anatomy of a patient, and then transfer this knowledge for the segmentation of planning fractions’ MRIs. Overall, there was no clear improvement over scenario ii; segmentation performance improved for some structures and degraded for others.

Contouring multiple OARs is time-consuming and somewhat subjective. The process requires going back and forth between slices multiple times to determine the shape of the organ. On average, based on our and others experience (18), it takes close to two hours to manually contour the organs for the treatment plan. Our proposed automatic segmentation takes <3.5 min for a dataset. As discussed above, we assume that the automatic contours in some cases will need expert refinement. Based on preliminary data, the time to adjust the network generated contours in MIM is about 30 min, making the procedure substantially shorter than two hours.

The study has several limitations. The small number of subjects limits the network’s performance. In fact, using the LOO approach, fourteen patients were used in the training of the fifteen networks in scenario i. In the future, a larger set of patients’ MRIs will be contoured to build the knowledge bank for the DL software.

In conclusion, our results demonstrate the promise of DL in volume segmentation of LACC. These developments provide a solid basis for the development of a robust auto-contouring tool to improve workflow efficiency and patient tolerance of the MRI-g-OART process.

## Data Availability Statement

The original contributions presented in the study are included in the article. Further inquiries can be directed to the corresponding author.

## Ethics Statement

The studies involving human participants were reviewed and approved by IRB ID: 20160817 at University of Miami. The patients/participants provided their written informed consent to participate in this study.

## Author Contributions

RS and LP contributed to conception and design of the study. LP and EM provided patient imaging for use in study. JF established the MR acquisition sequences. AB modified CNN code and performed experiments. OZ-R provided network expertise. JB, BS, LP, DA, NP, and RS organized patient database and provided original volumes. RS, IX, and MA performed statistical analysis. RS, AB, and BS wrote the first draft of the manuscript. RS, IX, and MA performed statistical analysis. All authors contributed to manuscript revision, read, and approved the submitted version.

## Funding

Research reported in this publication was supported by the National Cancer Institute of the National Institutes of Health under Award Numbers P30CA240139 and UO1CA239141.

## Author Disclaimer

The content is solely the responsibility of the authors and does not necessarily represent the official views of the National Institutes of Health.

## Conflict of Interest

The authors declare that the research was conducted in the absence of any commercial or financial relationships that could be construed as a potential conflict of interest.

The handling editor EA declared a past co-authorship with the author RS.

## Publisher’s Note

All claims expressed in this article are solely those of the authors and do not necessarily represent those of their affiliated organizations, or those of the publisher, the editors and the reviewers. Any product that may be evaluated in this article, or claim that may be made by its manufacturer, is not guaranteed or endorsed by the publisher.
